# Injury associated with methamphetamine use: A review of the literature

**DOI:** 10.1186/1477-7517-3-14

**Published:** 2006-03-29

**Authors:** Janie Sheridan, Sara Bennett, Carolyn Coggan, Amanda Wheeler, Karen McMillan

**Affiliations:** 1 School of Pharmacy, University of Auckland, Tel: 0064 9 373 7599 ext 85247, New Zealand; 2 Alcohol Advisory Council of New Zealand (ALAC), New Zealand; 3 Safe Communities Foundation, New Zealand; 4 Clinical Resource and Research Centre, Waitemata District Health Board, Auckland, New Zealand; 5 C/O University of Auckland, New Zealand

## Abstract

This paper reviews the literature exploring issues around methamphetamine and injury. There was a paucity of peer reviewed quantitative research and a lack of large scale epidemiological studies. Further sources described cases and others described injury risk as part of an overall review of methamphetamine misuse. Thus, a number of limitations and potential biases exist within the literature. The main areas where associations were noted or extrapolated with methamphetamine use and injury were around driving and violence. Other associations with injury related to methamphetamine manufacture. There was also circumstantial evidence for third party injury (that is injury to those not specifically involved in drug use or drug manufacture); however, the available data are inadequate to confirm these associations/risks.

## Introduction

Methamphetamine is a stimulant drug with high dependence liability, and is a commonly misused illicit substance worldwide. The drug first appeared as a legal drug in nasal decongestant preparations during the 1930s, but methamphetamine has been used for non-medical purposes wherever it has been readily available. Methamphetamine is a powerful psychostimulant which has a high dependence liability [1,2] and can be readily manufactured from precursor substances which are common ingredients of 'over the counter' cold remedies [3,4]. Methamphetamine can be smoked, snorted, injected or swallowed and its effects can last up to 12 hours.

The desired effects from the use of illicit methamphetamine include euphoria, decreased need for food and sleep, increased mental alertness and energy levels, disinhibition, sense of wellbeing and increased confidence [5]. Effects are dose-dependent and onset ranges from 20–40 minutes if swallowed, 3–5 minutes when snorted, and almost immediately if smoked or taken intravenously. Smoking and injecting users report an intensely pleasurable wave of sensation or 'rush' [6].

Repeated and chronic use of methamphetamine may result in undesirable and dangerous effects including psychotic symptoms such as severe paranoia, hallucinations and delusions of persecution, anxiety symptoms, agitation and affective symptoms such as depression and mood swings [3,7,8]. Other reported effects of chronic use include insomnia, malnutrition, involuntary movements, increased psychomotor activity, elevated blood pressure and heart rate sometimes resulting in myocardial infarction or stroke [9-12]. Continued use has a high dependence probability [4].

The most common symptoms of withdrawal from use are depression, [2,13] fatigue, restlessness, nervousness, feelings of alienation and irritation, craving for more methamphetamine, severe paranoia, hallucinations and delusions [14-16]. The physical effects of withdrawal can include tremor, sweating, headaches, dizziness, dry mouth and gastrointestinal problems. Severity of withdrawal depends on length and magnitude of use [5] and may be compounded by any pre-existing mental health problems [17].

The purpose of this paper is to provide a critical review of the English language peer reviewed and 'grey' literature on the association between methamphetamine use and injury.

### Literature reviewed for this paper

The Medline, Embase, PsychInfo, ERIC and Factiva databases were searched over the period 1997 to 2005 for references to injuries and other harms related to methamphetamine or "P' use. Key terms included: *methamphetamine, street drugs (ice, crank, pure or crystal meth)*, or illicit *drugs *combined with *injur$, harm$ *or *risk$*. Search terms varied slightly depending on terminology used in individual databases. In addition the websites of drug agencies such as the United National Office for Drugs and Crime (UNODC), the National Institute on Drug Abuse (NIDA), and the Australian Crime Commission were searched for relevant reports. Key studies were checked for further references. Additional material was sourced from those working in the drug misuse and control fields in New Zealand. The result was the creation of an Endnote database containing 174 references to research, information bulletins and reports and other relevant publications on the topic of methamphetamine or 'P' use and its potential for harm to the user and others, including children and emergency service workers.

Much of the available evidence on which this review has been based is of limited scope and variable quality in terms of gold standard research. In practice we found no population-based studies or large trials which provided insight into the burden of methamphetamine use and its association with injury. Whilst providing limited insight into some of the potential issues of injury associated with the use of methamphetamine, only limited conclusions can be drawn from the data.

### Injury associated with methamphetamine use

In news reports and public discussion both in New Zealand and internationally, methamphetamine use has been regularly implicated in deaths and injuries arising from violent crime, homicide, suicide, and police car chases [18-25]. Much of this association is speculative, inferred from the known effects of the drug on the body and on behaviour and from the conditions of manufacture of the drug [18,26]. Despite the circumstantial and anecdotal implications, the involvement of methamphetamine in injury (in particular with regard to violence) is not well understood, [27] and the literature offers only snippets of data on the type and rate of trauma injury and criminal harms associated with methamphetamine use.

Logan, Fligner and Haddix's review of Washington State deaths in which methamphetamine was detected in the blood of the deceased found the direct toxic effects of the drug caused or contributed to over a third of the deaths. Most of the methamphetamine deaths occurred at high blood concentrations of more than 0.5 mg/L. Death was also found to occur with concentrations as low as 0.05 mg/L – although usually in conjunction with other drugs or significant natural disease. A large proportion of the deaths were classified as homicide (27%) or suicide (15%) [28].

An examination of methamphetamine related deaths in Taiwan assigned 59% to accidental causes (including unintended overdose), with 14% homicidal, 11% suicidal and 13% natural causes (in the presence of a pathological illness). The majority of the fatalities were aged between 20–40 years and were predominantly male [29].

Evidence from the United States (US) indicates that in California, while methamphetamine use by trauma patients increased significantly over the period 1989–1994, cocaine rates remained constant and there was a decrease in alcohol rates [30]. Methamphetamine was most commonly associated with vehicular trauma, while cocaine positivity correlated with interpersonal violence. Furthermore, the mechanisms of injury associated with methamphetamine use in trauma patients resembled those associated with alcohol. The authors note that no causal relationship was found between methamphetamine use and trauma, and that results may be biased, as there was testing bias towards males, minorities, those involved in violence and those in car crashes.

A retrospective study which compared 461 patients with positive toxicology screens for methamphetamine and 32,156 others presenting at an emergency department in the US, found a significant association between methamphetamine use and injury from trauma among the methamphetamine-positive patients, the majority with blunt trauma injuries involving motor vehicle accidents. Other injuries involved interpersonal trauma resulting from gunshot wounds, stabbings and assaults. Again, the results may be biased due to selection bias in screening for drugs [31].

A study of trauma patients in Hawaii noted that patients testing positive for amphetamines were more likely to have self-harmed, were older and had stayed significantly longer in hospital than those who tested negative. They concluded that stimulant users utilised resources in the trauma centre which were "out of proportion to injury severity" [32].

Much of the study data on methamphetamine-related injury are derived from blood testing of emergency department users and the deceased. Significant testing bias may exist [30], and there may also be an underestimation of drug use as those who admit to using drugs are usually tested [31].

### Injury associated with motor vehicle use

The same propensities for risk-taking as well as those for paranoia, fearlessness and aggression might also be expected to have an adverse effect on driving behaviours [18,33]. There is some evidence that, at low doses in fatigued subjects, methamphetamine improves motor skills relevant to driving [18,34]. However, overall the evidence base concerning the impact of amphetamine-type stimulants on driving is equivocal, largely because there is little research evidence from which firm conclusions can be drawn [18]. A 1996 study of 28 cases of drivers arrested or killed in traffic accidents who tested positive for methamphetamine, concluded that methamphetamine "at any concentration is likely to produce symptoms that are inconsistent with safe driving" [35].

An Australian investigation into the incidence of alcohol and drugs in fatally injured drivers found that while alcohol was the drug involved most often in road crashes, its presence had decreased over the decade while cannabinoids and opiates had increased. Stimulants, mainly methamphetamine, had a much larger presence in the blood of truck drivers, than in car drivers or motorcyclists, who had been fatally injured [36]. In a further study by Longo and colleagues examining the relationship between substance use and culpability for 2,500 injured drivers, found that there was a higher proportion of stimulant-positive culpable drivers than drug free drivers, although the results were not statistically significant [37]. Drummer and colleagues' 10 year multi-centre study of drugs in drivers killed in Australia found that 4.1% (138) of the 3398 cases had stimulants in their blood, and that whilst only 3.4% of car drivers tested positive, 23% of truck drivers tested positive [38].

Another Australian study found that poly-drug use was common among traffic offence detainees – nearly a quarter tested positive to amphetamines (almost exclusively methamphetamine). Twenty percent tested positive to amphetamine plus another drug, and that other drug was most frequently cannabis [39]. As concomitant use of other drugs is likely to further affect judgement and ability to drive, the potential for drivers to be influenced by other drugs such as alcohol and cannabis in addition to amphetamine is considered to be a key issue by Australian Police [18].

The detection of drug-impaired drivers is far more difficult and complex than the detection of alcohol-impaired drivers [18], as blood concentration of methamphetamine is not a good measure of its effect on driving performance – particularly as drug withdrawal is as much a problem as drug intoxication [35,40]. Indeed, Logan describes a US case study of accidents involving methamphetamine-positive drivers where the drivers' behaviours leading to the accident were more consistent with methamphetamine withdrawal than intoxication [35].

One of the primary effects of methamphetamine is the forestalling of fatigue and Australian police are aware of the use of amphetamines in general by some long distance truck drivers [18]. The extent to which use of such substances contributes to crashes is unclear [18]. While higher methamphetamine blood concentrations may have harmful effects on self-perception, critical judgement, attention, risk taking, mood and motor restlessness, small amounts of amphetamines may improve driving performance in tired drivers [35].

The period following methamphetamine intoxication, when the direct effect is waning, is a period associated with fatigue, anxiety, irritability and 'microsleeps' [35]. These conditions are not conducive to safe driving [35], and may be a mechanism of crash causation [36]. The post-intoxication phase may also, in chronic cases, include delusions, hallucinations and suicidal ideation possibly impacting on driving.

With regard to injury or loss of life, high-speed car chases are among the riskiest of policing activities [18]. Whilst in New Zealand, as in Australia, there is currently no research evidence to indicate that offender use of methamphetamine is a significant factor in such pursuits, circumstantial evidence does suggest that methamphetamine use may increase both the likelihood and dangers of users becoming involved in high-speed police car chases [18].

Aitken and colleagues [41], in an Australian study using focus groups and a survey of 160 injecting drug users, also reported that methamphetamine users were very likely to link their drug use with driving – often driving for the 'thrill' of it. Evidence suggested some of the motivations for methamphetamine use, such as thrill seeking and the need for excitement, as well as the effects of amphetamine intoxication, may be both more conducive to engagement in a chase, and to other risky driving behaviours and 'more exciting' types of criminal activities. These behaviours may contribute to offenders being more likely to be pursued by police [18].

As with driving, there are also concerns with the use of other machinery either in the workplace or at home. For example, reports have suggested that methamphetamine is used within workplace settings to increase performance and endurance at work [42-44].

### Injury associated with violence

The combination of drugs and alcohol has long been regarded as a major risk factor for violence and crime, and the extent of drug use has been found to be a predictor of criminal activity [45]. Violence is not a psychopharmacological attribute of methamphetamine use, but as states of intoxication and withdrawal are often accompanied by disinhibition, agitation, paranoia, and delusions [5], these may lead to hostile feelings and violent behaviour [1,27]. In light of this, there are many aspects of the effects (aggression, paranoia, irrational behaviour and disinhibition) and patterns of methamphetamine use and the context of its manufacture that give rise to concerns about this particular drug's potential relationship with violent crime [46].

US law enforcement agencies have associated use of the potent forms of methamphetamine with levels of criminal violence far exceeding those experienced in relation to any other drug use and have suggested a potential relationship between methamphetamine use and 'thrill'-related crimes [47]. Klee and Morris [45], suggest that while the criminal activity of heroin users is often driven by the need for money to fund their drug use, the criminal activity of amphetamine users was motivated more by the need for excitement and thrills.

In Queensland, health workers have recently been experiencing increased instances of clients with paranoid and aggressive behaviours associated with methamphetamine use [48]. A particular population of concern are intravenous drug users who had previously been using heroin and had begun using methamphetamine during a period of 'heroin drought', and who exhibited clear deleterious changes in their mood and behaviour. The Queensland Ambulance Service also reported increased attendance at amphetamine-related cases over this period noting that they were very resource intensive because of the potential for the patient to be agitated and aggressive [48]. Methamphetamine-related aggressive behaviour is also an emerging issue of concern for law enforcement officers [49].

In a recent New Zealand study of drug enforcement officers' perceptions, two-thirds reported noticing changes in the level of violence being committed by methamphetamine users, although it is not possible to relate this to offences. Forty percent of those who reported change, reported noticing *"more serious violence"*, and 26% reported noticing *"more domestic violence" *[50]. Family and relationship breakdown is typically associated with drug dependence, and any aggression and violence resulting as a consequence of methamphetamine-induced paranoia, hostility and agitation is particularly likely to be manifest in domestic violence [3].

Methamphetamine users are not simply potential perpetrators, but also appear to be victims of violence. A study of 1016 methamphetamine-dependent individuals enrolled in a treatment programme in the US showed that suffering physical violence was reported by 85% of women and 70% of men [51], although the actual contribution of the drug to the violence experienced is difficult to assess.

### Injury related to manufacture

Manufacture of potent forms of methamphetamine involves relatively simple chemical processes [4,52,53]. Whilst simple, the manufacture of methamphetamine involves the use of highly flammable, very toxic and corrosive chemicals [54,55]. Injuries can occur through a range of methamphetamine manufacturing mechanisms. Exposure to toxic chemicals can occur via skin absorption and inhalation; burns, eye injuries, and poisoning may occur, and the injuries will depend on the method of manufacture [56], which will depend on the availability of precursor substances. Literature on the conditions of illicit manufacture and clandestine laboratory-related injuries is largely descriptive, or inferential.

Groups of people placed at risk are the methamphetamine manufacturers, other adults and children who may be resident where a laboratory is located, neighbours, later occupants of the site, police and forensic scientists [57]. Emergency workers may also incur some health risks if not adequately protected and there are wider environmental risks involved in the storage and disposal of highly toxic chemicals [54,57,58].

Many manufacturers may also be heavy methamphetamine users and have been reported to regularly take risks when handling dangerous chemicals and in the processes of manufacture [59,60]. In the US, one in five laboratories is discovered because of an explosion, and there is a risk of severe burns to anyone near a laboratory [55]. Case reports of patients involved in methamphetamine manufacture detail second-degree burns, and anhydrous ammonia ocular injury [61]. A retrospective study of case notes of 507 burns units admissions in 1999–2001 found 34 were either involved in the use of methamphetamine, or its production [62]. The authors also noted the cost of treatment was high, with average length of stay being around 16 days at a mean cost of $78,580 US. In a retrospective case series analysis of patients with facial burns admitted to a US burns unit, methamphetamine was believed to be responsible for almost 10% of those admitted [63]. A recent report to the Centers for Disease Control and Prevention (US) of notifications from 16 states to the Hazardous Substances Emergency Events Surveillance noted that 1,791 (4%) were associated with methamphetamine manufacture. Almost 1,000 people were injured – the most common injuries were respiratory irritation (39%), headache (26%), eye irritation (8%), and burns (8%) and included one fatality [64]. Methamphetamine manufacturing has also resulted in death by phosphine gas poisoning [65].

Special consideration must be given to environmental decontamination of methamphetamine laboratory sites and to the protection of exposed populations during this process [54]. There are high hazard risks associated with the closing down and deconstruction of a laboratory, and subsequent scene investigation. Law enforcement agents need specialist knowledge and equipment and ongoing training to safely dismantle idiosyncratic and unsafe laboratories [42]. The mobility and makeshift nature of clandestine laboratories exacerbates the dangers and increases the risks of harm to others. Methamphetamine laboratories have reportedly been found in sites as diverse as a motel room, a farm granary, a farm water tank and the back of a car [66-69].

There is potential for secondary contamination of emergency department (ED) personnel in cases where individuals contaminated by the processes of methamphetamine manufacture have presented at medical centres [57,70]. The risk of injury through secondary contamination depends on the toxicity and concentration of the substance on the patients' hair, skin and clothing, and the duration of contact that ED personnel have with the patient [70].

Disposal of chemical waste products from methamphetamine production creates human and environmental risk [71]. Illicit disposal is likely to be indiscriminate, such as directly into the ground, down drains and toilets – especially with the arrival of police – and concealed chemical stocks may be dangerously stored in unsafe containers [59]. The chemicals used and the gases produced can leach into and contaminate the fabric and furnishings of buildings, and an abandoned laboratory in a domestic residence poses risks to any unwitting future occupants [72-74].

### Injuries and children

Methamphetamine is easily and frequently manufactured in private residences, and there are reports of increasing numbers of children presenting to emergency centres in the US suffering the effects of exposure to methamphetamine in their homes [75]. The National Drug Intelligence Centre report on children at risk notes that 2028 children were present at seized methamphetamine laboratory sites in the US and that 35% of those tested positive for toxic levels of chemicals [76]. The report lists the direct injuries that children present at methamphetamine laboratories are exposed to, including inhalation, burns and more indirect injuries from neglect, malnutrition, and abuse [76].

Mecham and Melini [75] discuss toxicological and social consequences of children being present at methamphetamine laboratories, including caregiver neglect, access to weapons and targets of sexual abuse. Children exposed to methamphetamine also demonstrate respiratory distress, agitation and hyperactivity [75]. Other reported health effects included gastrointestinal problems, chemical burns, brain damage, headaches, and skin and eye irritation [77]. Kolecki's review of 18 paediatric patients [12], inadvertently poisoned with methamphetamine shows that they commonly presented with tachycardia, agitation, inconsolable crying and irritability, and vomiting. The more dependent a child is on its caregiver, the more consistently the passive exposure affects the child. Infants and toddlers in the home for long periods are likely to be the most vulnerable group.

### Synthesis of review

Robust data on injuries associated with methamphetamine use are scant and with substantial gaps in the available scientific knowledge. In part, whilst studies and case series suggest that methamphetamine use has been associated with behaviours that carry injury risk, causality is difficult to establish. In the absence of formal epidemiological analyses, confounding by other factors that may also be associated with methamphetamine use and injury has been inadequately addressed. Other limitations of the literature include measurement bias, lack of clear definition of injury and a lack of standardised approaches to measurement.

In evaluating the current literature on injury associated with methamphetamine use, it is also essential to note international differences, e.g. in route of administration, user subgroups and functional use, and use patterns with other substances. Furthermore, study populations and data sources commonly accessed and relevant tended to come from particular populations. The clinical research sourced predominantly originated in the US and much is based on work undertaken by treatment organisations with an established cocaine treatment background. Thus attention is often directed at cocaine-derived issues. Also samples taken from those in treatment cannot be expected to be representative of the general population and many subjects have had a history of other drug and/or intravenous drug use.

Similarly the studies undertaken in Australia have largely been undertaken by organisations funded to research HIV and therefore tend to focus on the use of methamphetamine among men who have sex with men (MSM) (the largest HIV risk group in Australia) and in particular MSM in relation to high risk sexual practices. This tends to present what may be a misleading picture of methamphetamine as a 'gay' drug in Australia.

A number of links that have been made, such as those between unsafe driving and methamphetamine use, criminal violence and methamphetamine use, are largely inferential and based on what is known about the psychophysiological effects of methamphetamine. Much of the police data are reducible to a single source or set of sources that have been recycled through Australian and New Zealand documents.

Research outputs from hospital emergency departments often use drug testing of blood samples to validate clinical reporting. However, universal blood testing of patients at sites providing emergency care is limited by ethical and practical considerations, and emergency department provider decisions about who to test have been shown to be subject to bias – with males, ethnic minority groups and those involved in motorcycle crashes being more likely to be tested. As long as the biases remain constant this may be a reliable indicator of changes of rates, but not of overall levels of use.

Data collection from emergency departments could provide a valuable insight into violence, injuries, issues around child welfare and domestic violence. However, it is difficult to ensure that the idiosyncratic aspects of routinely collected data within emergency departments do not undermine opportunities to collect data. A more feasible form of data collection is likely to be researcher initiated and to be prospective in nature. Such data could inform the development of interventions designed to reduce risk associated with both methamphetamine use and methamphetamine manufacture, in particular in relation to children and domestic violence. Testing of patients would need to be conducted both for use of methamphetamine and for contaminants associated with manufacture.

Further research into the burden of injury associated with methamphetamine use and driving requires attention and includes road traffic incidents associated with police car chases. More qualitative investigation of the function of methamphetamine use among drivers, as well as the impact the drug has on ability to perform complex physical and cognitive actions will provide information for the development of interventions.

One of the most common causes of injury associated with methamphetamine use is likely to be violence. Police arrest and conviction data might be utilised to target interventions at individuals as well as to aid in exploration of the setting associated with such events. However, routine collection of data from emergency departments, police, environmental health and other related organisations is costly, time consuming and often impractical. Ultimately, engagement and consultation with the various agencies and the co-operation of those agencies and their staff is essential to any mobilisation of the will to institute procedures that would further the collection of useful data.

Of particular concern is the potential impact of methamphetamine-related injury risk on child welfare. It may be prudent to begin to explore injury prevention and harm reduction interventions relating to this, in parallel with any research and data collection. Raising awareness of the potential adverse effects of methamphetamine use and manufacture on children is an urgent issue.

There is also a case to be made for closer attention to be paid to the specificities of methamphetamine itself with regards to particular locations and situations in which it is used, as to a certain extent, a more general approach may overlook important aspects of the drug, such as its effects and its particular methods and patterns of use. This would require not only further targeted epidemiological study, but also more ethnographic research that might further contribute to an understanding of the social practises of methamphetamine use, the meanings attached to it and the social contexts in which use occurs.

## Competing interests

The author(s) declare that they have no competing interests.
